# Identification of key genes and immune infiltration of diabetic peripheral neuropathy in mice and humans based on bioinformatics analysis

**DOI:** 10.3389/fendo.2024.1437979

**Published:** 2024-11-18

**Authors:** Yumin Zhang, Hui Zhou, Juan Liu, Nan Zhou

**Affiliations:** ^1^ Department of Geriatric Endocrinology, Jiangsu Province Hospital and Nanjing Medical University First Affiliated Hospital, Nanjing, China; ^2^ Department of Orthopedics, The First Affiliated Hospital of Zhengzhou University, Zhengzhou University, Zhengzhou, China

**Keywords:** diabetic peripheral neuropathy, bioinformatics analysis, differentially expressed genes, hub genes, immune infiltration

## Abstract

**Background:**

Diabetic peripheral neuropathy (DPN) is a common chronic complication of diabetes, while the underlying molecular mechanisms are still unclear. The aim of this study was to screen the key genes and the roles of immune infiltration in DPN using bioinformatics analysis.

**Methods:**

DPN mice datasets including GSE222778, GSE11343, GSE70852, GSE27382, and GSE34889 were retrieved from the GEO database. Data of human DPN were retrieved from the dbGaP. The differentially expressed genes (DEGs) were selected and further analyzed by using Gene Ontology, Kyoto Encyclopedia of Genes and Genomes enrichment analysis, and Gene Set Enrichment Analysis (GSEA) to find the shared key pathway. Protein–protein interaction networks were built in shared mouse and human DEGs. The hub genes were selected and verified *in vitro* using high- glucose-treated PC12 cells and Schwann cells. The single-sample GSEA (ssGSEA) algorithm was used to analyze the proportions of infiltrating immune cells in human DPN and the subsequent correlations with hub genes.

**Results:**

A total of 323 mouse DEGs and 501 human DEGs were selected, and they were found significantly enriched in immune-related biological functions and pathways. A total of 13 DEGs were found shared in mice and human DPN datasets, and among them, there were 7 hub genes, namely, PLAUR, S100A8, IL7R, CXCL13, SRPX2, CD300LB, and CFI. The expression of Cfi, S100a8, Cxcl13, and Cd300lb was consistently confirmed *in vitro*. The scores of neutrophils and NK CD56bright cells varied most significantly by immune cell infiltration analysis (*p* < 0.01). Furthermore, the selected hub genes were found to be highly correlated with the immune infiltration.

**Conclusion:**

Our study indicated the importance of immune dysregulations in DPN and identified several hub genes through combined analysis in mice and human DPN samples, thus providing potential diagnostic and therapeutic targets in the future.

## 1 Introduction

Diabetic peripheral neuropathy (DPN) is a prevalent chronic complication of diabetes mellitus (DM), affecting approximately 50% of DM individuals in the world (1). Previous studies indicated that the prevalence of clinically diagnosed DPN could rise to 60% to 75% when more sensitive nerve conduction testing methods are conducted (2–4). DPN had brought up huge economic and medical burden for the affected individuals (5), as it served as a primary factor in the development of diabetic foot and was a significant contributor to non-traumatic lower limb amputations (6). Nevertheless, the initial symptoms of DPN were currently not apparent, and the relevant reliable biomarkers for early identification of DPN were scarce, which easily led to the affected patients progressing to an irreversible stage before clinical detection.

Prior research had shown that the onset and progression of DPN were primarily attributed to elevated blood glucose levels, insulin insufficiency, and abnormal lipid profiles (7, 8). However, the precise molecular pathways underlying nerve dysfunction and diminished regenerative potential remained poorly understood. The emergence of bioinformatics has introduced novel approaches to investigating DPN. Through the analysis of multi-omics data encompassing the genome, transcriptome, proteome, and metabolome, bioinformatics analysis could elucidate the molecular mechanisms and pathological pathways of the diseases, identify pivotal factors driving their progression, and establish a theoretical framework for personalized therapeutic interventions (9, 10). Furthermore, leveraging data mining techniques and machine learning algorithms, bioinformatics analysis could sift through vast biomedical datasets to pinpoint biomarkers associated with the onset, progression, and prognosis of defined diseases.

In recent years, there has been several studies conducted on the regulatory pathways and genes associated with DPN using bioinformatics analysis (11–19). For instance, Elzinga et al. employed a combination of the streptozotocin-induced db/+ murine model of type 1 diabetes mellitus (T1DM) and the db/db murine model of type 2 diabetes mellitus (T2DM) and found the crucial role of inflammation in the development of DPN (14). Li et al. utilized bioinformatics and machine learning techniques to identify potential biomarkers in DPN mice, ultimately pinpointing LTBP2 and GPNMB as diagnostic markers for DPN (19). Hal et al. used transcriptome analysis in human sensory neuron samples with DPN and found increased expression levels of inflammation-related genes and decreased expression levels of neuronal-related genes, thus revealing the contributions of inflammation and neuronal loss to pain on DPN (20). However, the previous studies primarily focused on either mice or human samples and seldom studies aimed to found conservatively shared pathogenesis among mice and human DPN. This study involved the acquisition of five datasets containing sciatic nerve (SCN) samples from mice with DM and a dataset containing dorsal root ganglia from human DPN. Differentially expressed genes (DEGs) were initially identified within these datasets. The enrichment pathways and biological functions of these shared DEGs were subsequently analyzed, which highlighted the significance of immune regulation in DPN, prompting further analysis of immune infiltration using the single- sample Gene Set Enrichment Analysis (ssGSEA) algorithm. Following this, the mouse DEGs were mapped to human gene IDs and validated in human DPN. The protein–protein interaction (PPI) networks were constructed using shared DEGs identified from mice and human DPN datasets to identify hub genes. MicroRNAs and transcription factors (TFs) associated with these shared DEGs were investigated. Additionally, the relationship between the hub genes and immune infiltration was examined. Finally, the mRNA expression levels of the hub genes were validated using a high- glucose-treated PC12 cell model *in vitro*. Overall, our findings underscored the significance of immune dysregulation in the pathogenesis of DPN and found key shared genes among humans and mice of DPN, which might serve as potential diagnostic and therapeutic biomarkers for DPN in the future.

## 2 Materials and methods

### 2.1 Collection of the datasets

**Figure 1:**
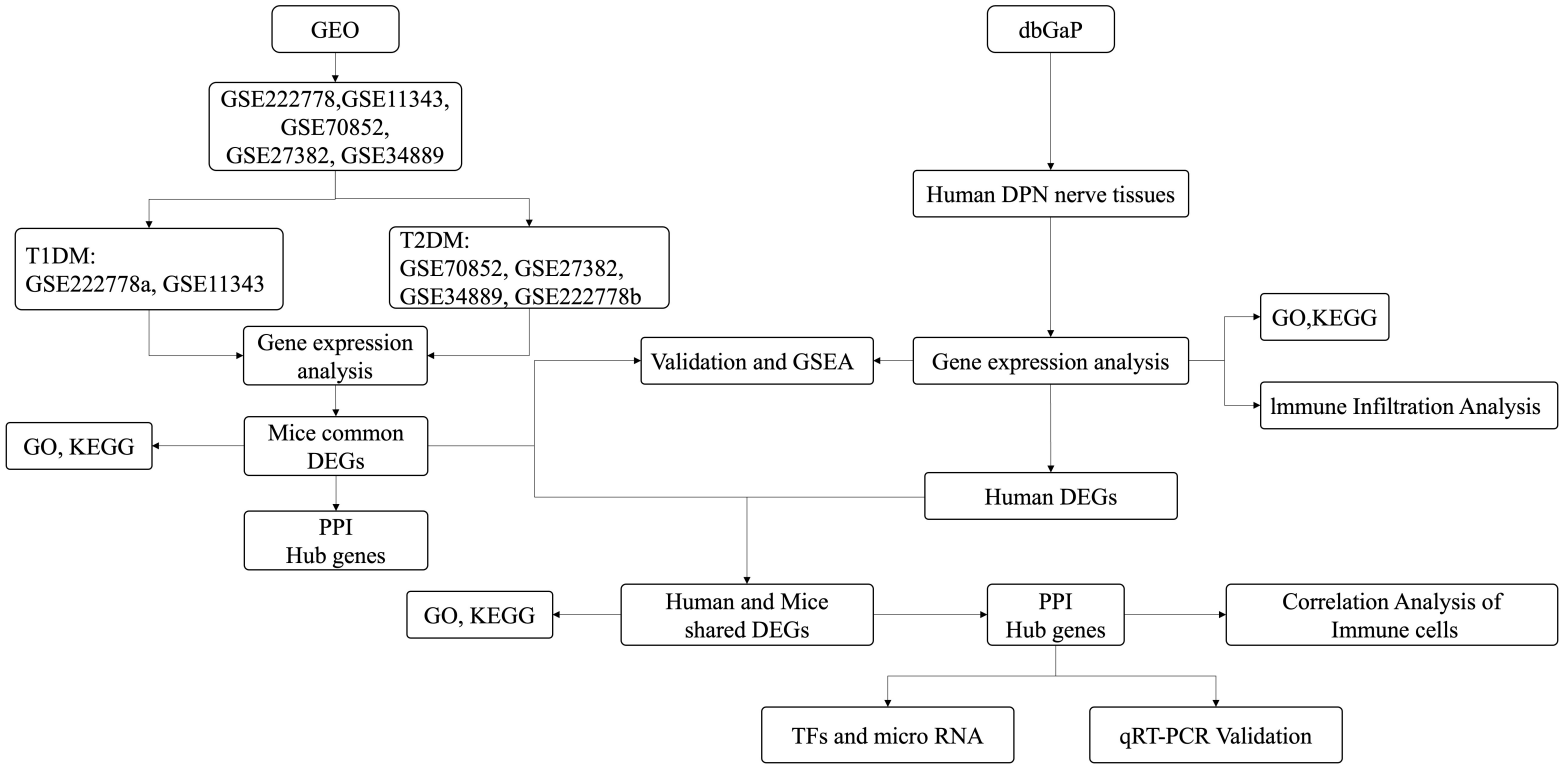
Flow chart of this study.

GSE222778, GSE11343, GSE70852, GSE27382, and GSE34889 were retrieved based on the keyword “diabetic peripheral nephropathy” and downloaded from the GEO database (https://www.ncbi.nlm.nih.gov/geo/). To ensure consistency of analysis, mouse samples only containing the SCN tissues were selected. The probes in the above datasets were subsequently annotated according to the platform file. **Table 1** shows the basic information of the above five datasets from mice and **Figure 1** shows the basic flow chart of the whole analysis. Microarray measurements including SCN samples of 16-week-old db/+, STZ-treated db/+, and db/db mice from the dataset GSE222778 were selected (*n* = 6 in each group). The dataset GSE222778 consists of two subgroups, one of which was STZ-induced T1DM mice and another was T2DM db/db mice; thus, we divided it into two groups, which we defined as GSE222778a and GSE222778b separately for further analysis. The GSE27382 dataset contains six SCN samples from 24-week-old db/db mice and seven SCN samples from db/+ mice. The GSE70852 dataset contains five SCN samples from 26-week-old ob/ob mice and five SCN samples from db/+ mice. The GSE11343 dataset contains four SCN samples from 24-week-old DBA/2J mice and four SCN samples from STZ-treated age-matched DBA/2J mice. The GSE34889 dataset contains seven SCN samples from 24-week-old db/+ mice and eight SCN samples from db/db mice. The original data of human diabetic peripheral nerve tissues were retrieved from the dbGaP with accession code phs002548.v1.p1, which includes five dorsal root ganglia samples of DPN patients and seven dorsal root ganglia samples of non-DPN patients.

**Table 1:** Overview of mice datasets with their GEO features and DEGs.

DM type	Strain	Age	GEO accession	GEO platform	Total DEGs	Upregulated DEGs	Downregulated DEGs
Type 1	db/+	16 weeks	GSE222778a	GPL13112	731	421	310
	DBA/2J	24 weeks	GSE11343	GPL1261	308	175	133
Type 2	ob/ob	26 weeks	GSE70852	GPL16368	161	61	100
	db/db	24 weeks	GSE27382	GPL9746	1,213	653	560
	db/db	24 weeks	GSE34889	GPL9746	1,233	669	564
	db/db	16 weeks	GSE222778b	GPL13112	1,035	370	665

### 2.2 Identification of differentially expressed genes

Six DPN mice groups in total were selected for further analysis including two groups of T1DM and four groups of T2DM. The GEOquery package in R (21) was used to download the original data of GSE11343 and GSE222778. The limma package was subsequently used to normalize the data and find the DEGs. DEGs for GSE70852, GSE27382, and GSE34889 datasets were analyzed through the GEO2R (https://www.ncbi.nlm.nih.gov/geo/geo2r/) web tool that also used the limma package for identifying DEGs. The Benjamini–Hochberg false discovery rate (FDR) method was applied to discover genes that were statistically significant and limited false positives. Genes that met the cutoff criteria, adjusted *p*-values (*p*_adj_) <0.05, and |log2FC| ≥1 were considered as DEGs in these six groups except for GSE11343, which had limited numbers of DEGs under the above criteria; thus, we reset the cutoff criteria, at *p*-values <0.05 and |log2FC| ≥1 instead. A volcano plot was used to visualize the differential analysis results. Venn analysis was used to find the common DEGs. Genes that have appeared three or more times in six groups of mouse DPN datasets were considered to be the common mouse DEGs.

### 2.3 Gene Ontology and pathway enrichment analysis of DEGs

Gene Ontology (GO) and Kyoto Encyclopedia of Genes and Genomes (KEGG) enrichment analysis were performed using the clusterProfiler package, org.Hs.eg.db package, enrichplot package, and ggplot2 package in R language for analysis and image generation separately. The significance cutoff value was set at a *p*_adj_ < 0.05.

### 2.4 PPI networks and the extraction of hub genes

The retrieval of interacting genes database called STRING (https://www.string-db.org/) was used to construct the PPI network of proteins derived from DEGs. The medium confidence score of 0.500 was set to generate the PPI network. The Cytoscape (v.3.9, https://cytoscape.org/) software was used for a visual representation and further PPI network studies. Cytohubba (https://apps.cytoscape.org/apps/cytohubba) and Molecular Complex Detection (MOCODE), two plugins in Cytoscape, were used to calculate the hub genes in the PPI network. Topological analysis including maximal clique centrality (MCC) was also selected for finding the top hub genes. The GeneMANIA (https://genemania.org) website was used to construct the hub gene network diagrams and present the relationships between the hub genes.

### 2.5 Identification of TFs and miRNAs

The NetworkAnalyst platform (https://www.networkanalyst.ca/) was used to construct TF–DEG and DEG–miRNA regulatory networks to analyze relevant TFs and miRNAs. The TF–DEG network was established using the JASPAR database. The DEG–miRNA network was established using the TarBase v8.0. In general, the degree filter was set as 2 to generate the optimal layout.

### 2.6 Gene Set Enrichment Analysis

The Gene Set Enrichment Analysis (GSEA) was performed using the clusterProfiler package (5, 22). The reference gene set was c2.cp.all.v2022.1.Hs.symbols.gmt [All Canonical Pathways] (3050). The significant conditions were *p*_adj_ < 0.05 and FDR (*q*-value) < 0.25. The ssGSEA algorithm provided in the R package-GSVA was utilized to calculate the immune infiltration status of the uploaded data (23).

### 2.7 Quantitative real-time PCR

The PC12 cell was obtained from the American Type Culture Collection and cultured in Dulbecco’s modified Eagle’s medium (DMEM) (PM150210B, Pricella, China). Generally, PC12 cells were cultivated in a medium with normal glucose (25 mM, D-glucose; NG) or high glucose (100 mM, D-glucose; HG) according to the previous literature (24) for 48 h. The Schwann cell RSC96 was obtained from Procell Life Science & Technology Co., Ltd. (CL-0199, China) and cultured in DMEM. RSC96 cells were cultivated in a medium with normal glucose (25 mM, D-glucose) or high glucose (50 mM, D-glucose) for 48 h (25). Total RNA was extracted from cultured cells with the TRIzon Reagent (CW0580S, CWBIO, China) and reverse transcription was performed to gather cDNA. The real-time PCR reactions were conducted using the SYBR Green PCR system (E096-01A, Novoprotein, China). The PCR program was set as follows: 95°C for 1 min, 95°C for 20 s, 55°C for 20 s, and 72°C for 30 s, for 40 cycles. Relative quantities were calculated using the 2^−ΔΔCt^ method with actin as inner control. The primers used for PCR are shown in **Supplementary Table 1**.

### 2.8 Statistical analysis

Data were expressed as the mean ± SEM. Principal component analysis (PCA) was performed to view sample differences after dimensionality reduction of high-dimensional data between the DPN group and the control group. Spearman correlation analysis was used between the immune infiltration scores and hub genes. The Welch *t* -test was performed for comparisons between the control group and the DPN group. *p*-values < 0.05 were considered statistically significant.

## 3 Results

### 3.1 Identification of DEGs and common DEGs among DPN mice

**Figure 2:**
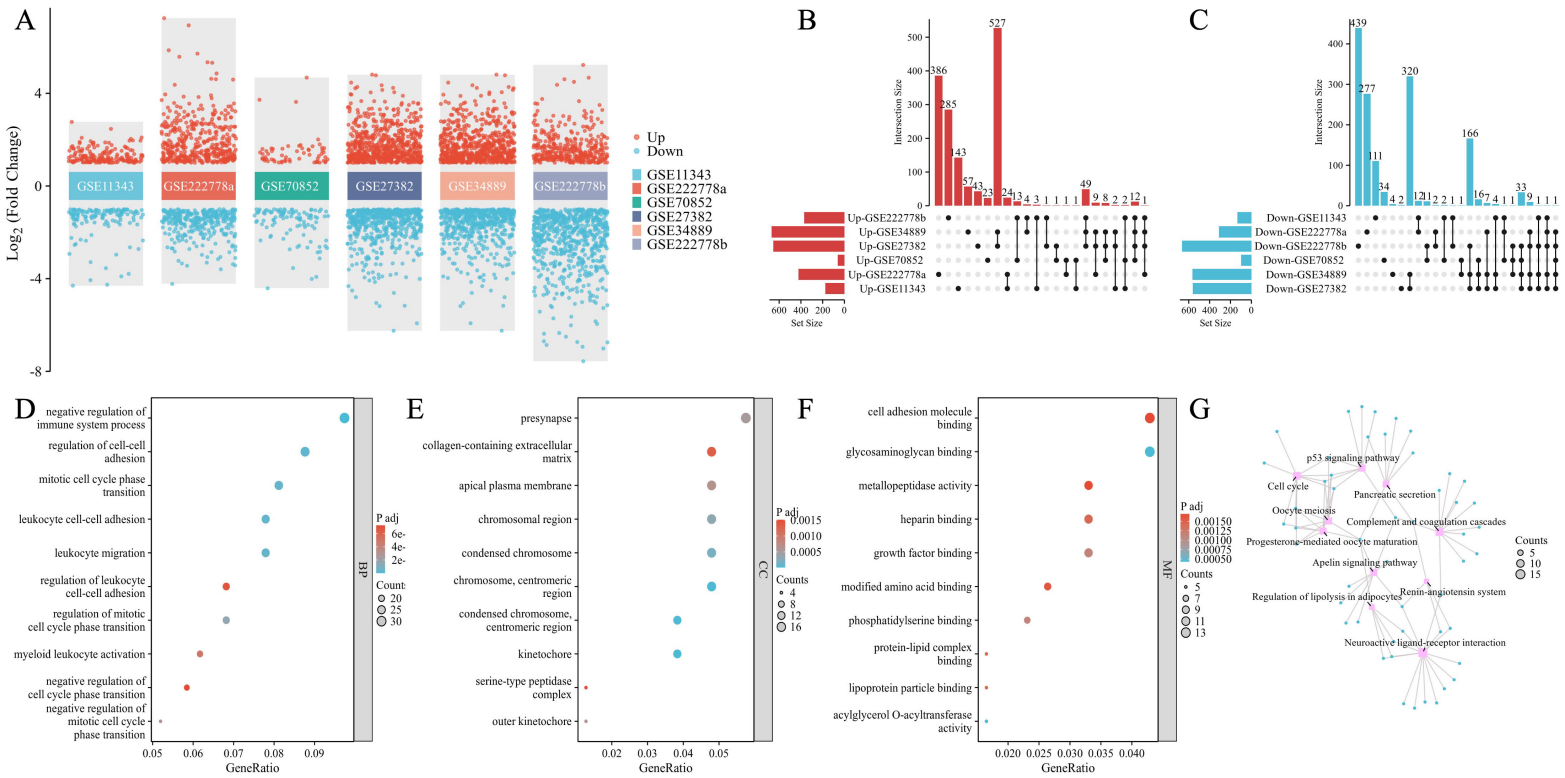
Identification of common DEGs and the functional enrichment and pathway analysis in DPN mice. **(A)** Dysregulated genes in six groups with significant differences. The red dots indicated upregulated genes and the blue dots indicated downregulated genes. **(B, C)** The UpSet plot of genes that were upregulated **(B)** and downregulated genes **(C)** in six groups. **(D–G)** GO annotations of common DEGs: BP **(D)**, CC **(E)**, MF **(F)**, and KEGG pathway **(G)**. Top 10 GO terms were illustrated.

As shown in **Table 1**, 731 DEGs (421 upregulated and 310 downregulated) were found in GSE222778a and 1,035 DEGs (370 upregulated and 665 downregulated) were found in GSE222778b. For GSE11343, there were 175 upregulated DEGs and 133 downregulated DEGs. In addition, 161 DEGs (61 upregulated and 100 downregulated) in GSE70852, 1,213 DEGs (653 upregulated and 560 downregulated) in GSE27382, and 1,233 DEGs (669 upregulated and 564 downregulated) in GSE34889 were found. The volcano plot in **Figure 2A** visually demonstrated the overall picture of gene expressions in the six groups, where red and blue dots indicated upregulated and downregulated genes with significant differences. Subsequently, the UpSet plot was utilized to select the upregulated and downregulated DEGs as shown in **Figures 2B, C**, and a total of 323 genes that have appeared three or more times in six groups of DPN datasets were considered to be the common mouse DEGs.

### 3.2 Functional enrichment and pathway analysis in common mouse DEGs

As shown in **Figures 2D–F**, the top 10 GO terms were illustrated in the bubble graphs for biological process (BP), cell compartment (CC), and molecular function (MF) respectively. The DEGs were significantly enriched in the negative regulation of the immune system process, regulation of cell–cell adhesion, mitotic cell cycle phase transition, leukocyte cell–cell adhesion, leukocyte migration, regulation of leukocyte cell–cell adhesion, regulation of mitotic cell cycle phase transition, myeloid leukocyte activation, negative regulation of cell cycle phase transition, and negative regulation of mitotic cell cycle phase transition in the subset of BP as shown in **Figure 2D**. In **Figure 2E**, the DEGs were significantly enriched in the presynapse, collagen-containing extracellular matrix, apical plasma membrane, chromosomal region, condensed chromosome, chromosome, centromeric region, kinetochore, serine-type peptidase complex, and outer kinetochore in the subset of CC. In the subset of CC, the DEGs were enriched in cell adhesion molecule binding, glycosaminoglycan binding, metallopeptidase activity, heparin binding, growth factor binding, modified amino acid binding, phosphatidylserine binding, protein–lipid complex binding, lipoprotein particle binding, and acylglycerol O-acyltransferase activity as seen in **Figure 2F**. The KEGG pathway analysis as shown in **Figure 2G** revealed the following top 10 pathways: complement and coagulation cascades, p53 signaling pathway, cell cycle, progesterone-mediated oocyte maturation, oocyte meiosis, neuroactive ligand–receptor interaction, apelin signaling pathway, regulation of lipolysis in adipocytes, pancreatic secretion, and renin–angiotensin system. **Supplementary Table 2** lists details of the top 10 GO terms and the KEGG enrichment pathways.

### 3.3 The immune dysregulation in human DPN peripheral nerve tissues by bioinformatics analysis

**Figure 3:**
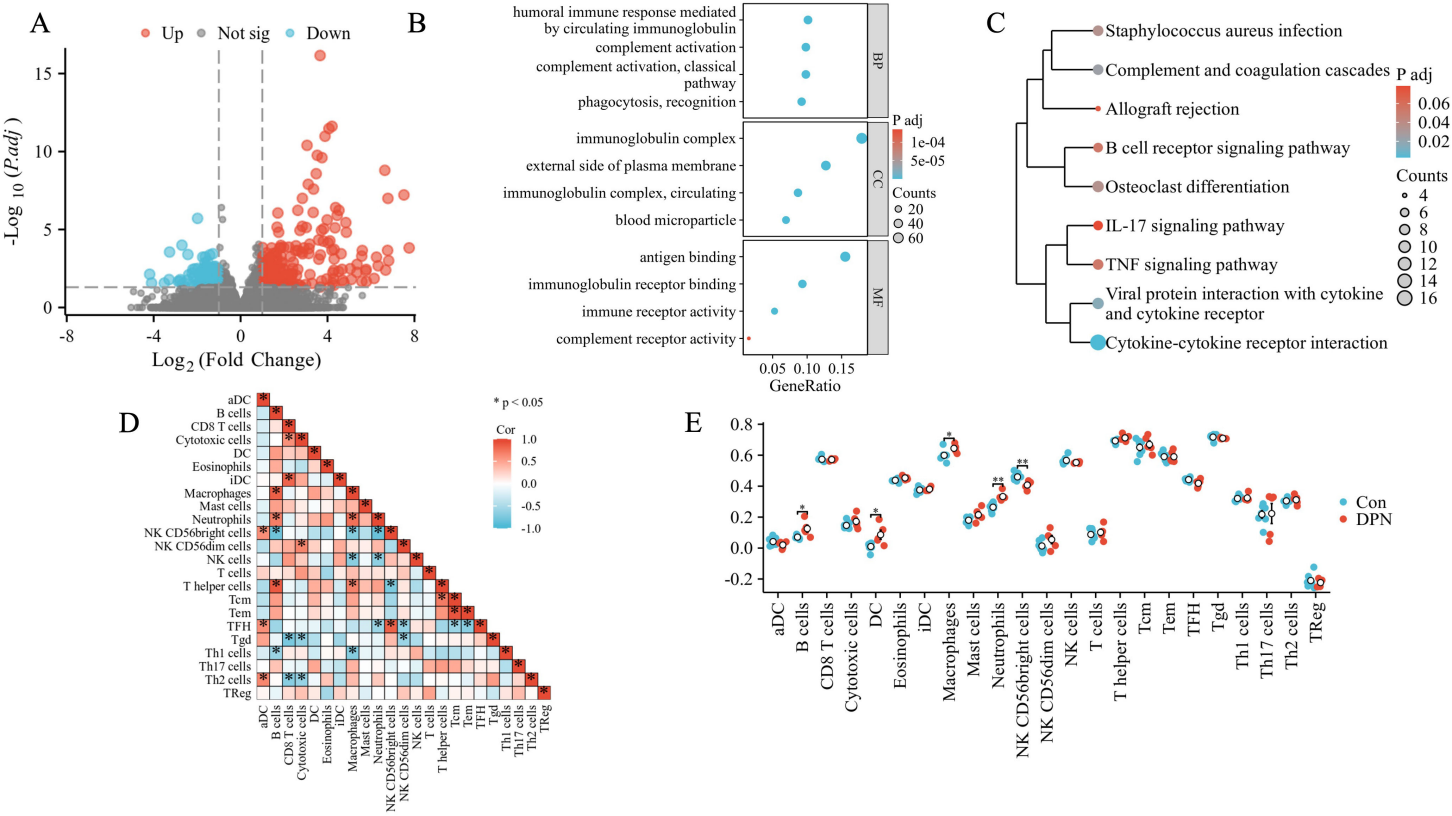
Bioinformatics analysis of human DPN peripheral nerve tissues. **(A)** Volcano plot of the distributions of all DEGs. The red dots indicated upregulated genes and the blue dots indicated downregulated genes. **(B)** GO annotations of DEGs. The top four GO terms were illustrated. **(C)** KEGG enrichment analysis of DEGs. **(D)** The correlation analysis of immune cells in DPN samples. Different shades of squares represent the degree of negative or positive correlation. **(E)** Comparison of the scores of immune infiltrations between control and DPN groups. *p < 0.05; **p < 0.01.

In order to validate our findings of DPN mice in human, we firstly downloaded data from the dbGaP with accession code phs002548.v1.p1, which included five dorsal root ganglia samples of DPN patients and seven dorsal root ganglia samples of non-DPN patients. A total of 501 DEGs were found as seen in **Figure 3A**, and subsequent functional enrichment analysis revealed that the DEGs were most significantly enriched in humoral immune response mediated by circulating immunoglobulin in the subset of BP, enriched in the immunoglobulin complex in the subset of CC, and enriched in antigen binding in the subset of MF as shown in **Figure 3B**. As shown in **Figure 3C**, the KEGG pathway analysis revealed the following nine pathways: cytokine–cytokine receptor interaction, viral protein interaction with cytokine and cytokine receptor, complement and coagulation cascades, *Staphylococcus aureus* infection, osteoclast differentiation, B-cell receptor signaling pathway, TNF signaling pathway, allograft rejection, and IL-17 signaling pathway, which were mainly involved in immune regulation. Subsequently, we did a GSEA in the human DPN samples as shown in **Supplementary Figure 1** and **Supplementary Table 3**. The top five pathways and biological processes were FCGR3A-mediated IL10 synthesis, signaling by the B-cell receptor BCR, Fcgamma receptor (Fcgr)-dependent phagocytosis, Fceri-mediated Nf-kB activation, and parasite infection as shown in **Supplementary Figure 1**, which were highly related to immune response. As the above analysis indicated that immune dysregulation played an important role in DPN, an immune cell infiltration analysis was utilized. As shown in **Supplementary Figure 2** and **Figure 3D**, the PCA cluster plot of immune cell infiltration and the correlation analysis of 24 immune cell types were separately presented. The scores of immune infiltration analysis were compared between the control group and the DPN group as shown in **Figure 3E**. Five types of immune cells were significantly different between DPN and control groups, namely, B cells (*p* < 0.05), DC cells (*p* < 0.05), macrophages (*p* < 0.05), neutrophils (*p* < 0.01), and NK CD56bright cells (*p* < 0.01).

### 3.4 Validation of mouse DEGs in human DPN

**Figure 4:**
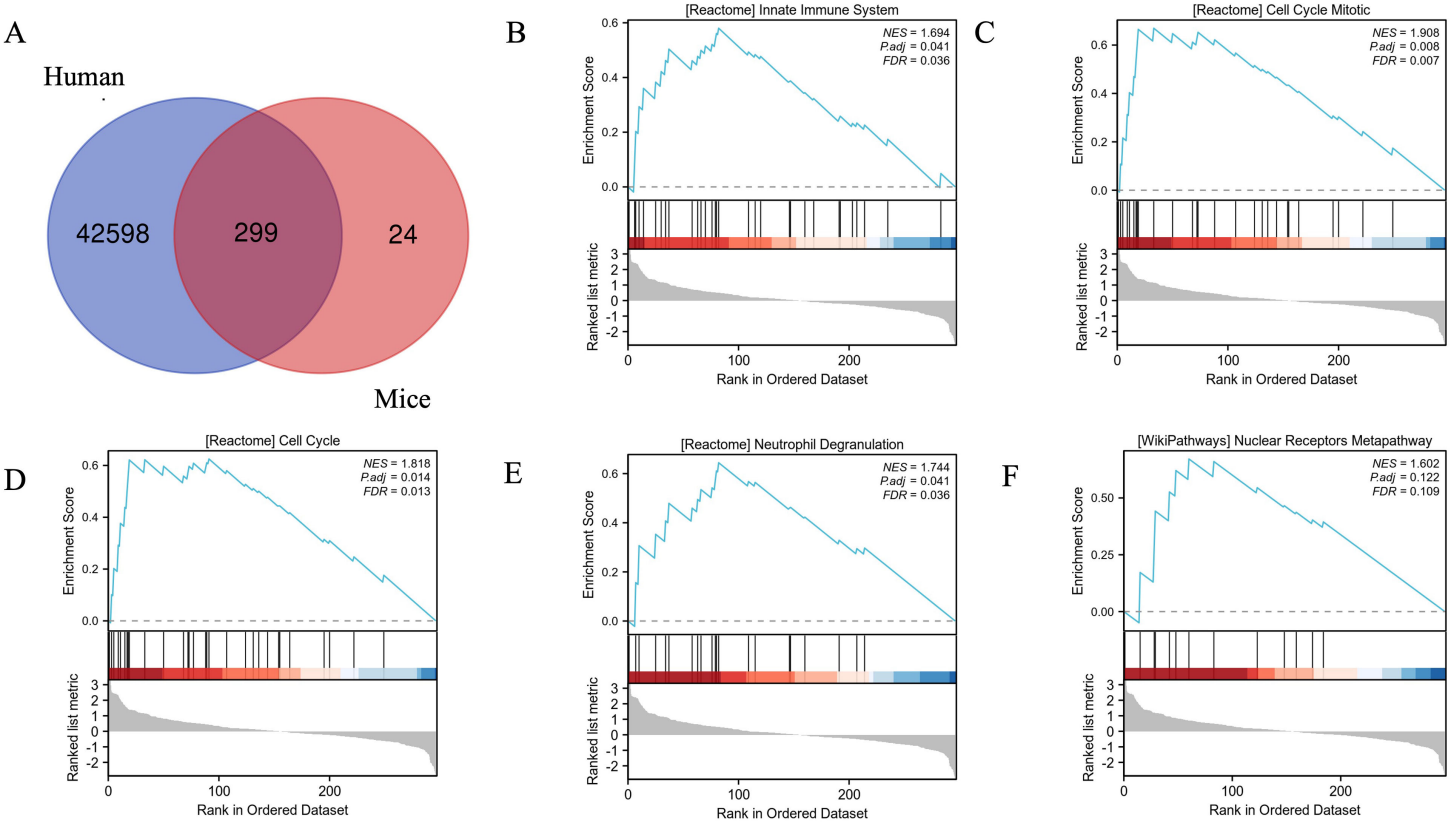
Validation of mouse DEGs in human DPN and the enrichment plots from GSEA. **(A)** The Venn diagram showed the validation of mouse DEGs in human DPN. **(B–F)** The enrichment plots from GSEA. Several pathways and biological processes were differentially enriched in cell cycle mitotic **(B)**, cell cycle **(C)**, neutrophil degranulation **(D)**, innate immune system **(E)**, and nuclear receptors meta pathway **(F)**.

In order to show the shared pathways of mouse DEGs in human DPN, we used the gene database from the NCBI website (https://www.ncbi.nlm.nih.gov/gene) to convert the mouse genes into human genes. In total, the intersection contained 299 genes (**Figure 4A**). Further GSEA showed that the top five enriched pathways were the cell cycle mitotic, cell cycle, neutrophil degranulation, innate immune system, and nuclear receptors meta pathway in human DPN (**Figures 4B–F**).

### 3.5 Intersection and functional enrichment analysis of human and mouse shared DEGs in DPN

**Figure 5:**
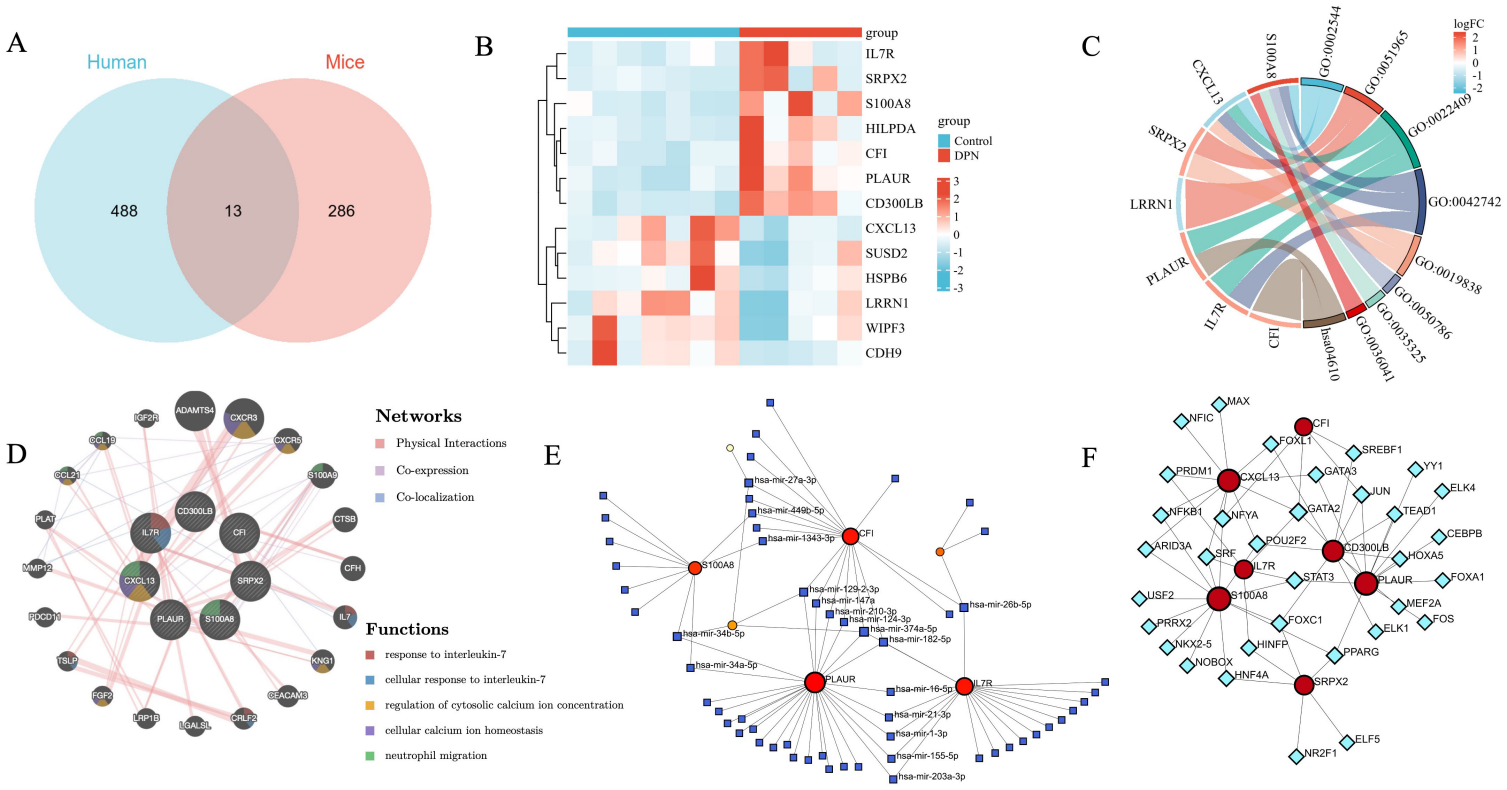
Co-analysis of mouse DEGs and human DEGs in DPN. **(A)** Venn diagram intersected mouse DEGs and human DEGs. **(B)** Heatmap displayed expression patterns of common DEGs in control and human DPN samples. **(C)** The Circos plot of enriched GO terms based on common DEGs. GO:0002544: chronic inflammatory response; GO:0022409: positive regulation of cell–cell adhesion; GO:0045785: positive regulation of cell adhesion, GO:0022407: regulation of cell–cell adhesion; GO:0002920: regulation of humoral immune response; GO:0019838: growth factor binding; GO:0048306: calcium-dependent protein binding; hsa04610: complement and coagulation cascades. **(D)** The PPI network of common DEGs generated by GeneMANIA. **(E)** TFs–DEG network. **(F)** DEG–miRNA network.

We further merged the mouse DEGs and human DEGs in DPN using Venn analysis, and as shown in **Figure 5A**, 13 genes were found, namely, HSPB6, LRRN1, CXCL13, SUSD2, CFI, CDH9, SRPX2, CD300LB, WIPF3, IL7R, HILPDA, S100A8, and PLAUR. The expression heat map of the human and mouse shared DEGs is illustrated in **Figure 5B**. As shown in **Figure 5C** and **Supplementary Table 4**, the top four GO terms of BP and MF included chronic inflammatory response, positive regulation of synapse assembly, positive regulation of cell–cell adhesion, defense response to bacterium, growth factor binding, RAGE receptor binding, and Toll-like receptor binding long-chain fatty acid binding. There was no significant CC subset, and only one KEGG pathway, the complement and coagulation cascades, was found.

### 3.6 Exploration of hub genes

We further generated the PPI network of the shared DEGs between human and mouse DPN. We used the online database STRING to calculate relationships between genes and introduced the interacting genes into Cytoscape for network visualization analysis as shown in **Supplementary Figure 3**. Seven hub genes were selected, namely, PLAUR, S100A8, IL7R, CXCL13, SRPX2, CD300LB, and CFI. The PPI network of the above hub genes included seven nodes and eight edges, which were visualized by GeneMANIA (**Figure 5D**).

In addition, the PPI network of mouse common DEGs were also established as seen in **Supplementary Figure 4A**. The plugin application MCODE was used to identify the core subnetwork (**Supplementary Figure 4B**). Additionally, the MCC algorithm in the plugin application “cytoHubba” was utilized to select the top 10 genes in the network, and completely overlapped with the gene MCODE selected (**Supplementary Figure 3C**), namely, Kif4, Kif20a, Cdk1, Cdca5, Ccna2, Bub1, Aurkb, Ttk, Ndc80, and Kif11.

### 3.7 Construction of regulatory networks

The interactions of miRNAs regulators with hub genes are shown in **Figure 5E**. Blue squares represented miRNAs and red circles represented hub genes. Our results showed that PLAUR, IL7R, CFI, and S100A8 were the top four genes with the highest scores of degrees and betweenness of this network. Moreover, we also detected the significant hub miRNAs from the miRNA–gene interaction network, namely, hsa-mir-374a-5p, hsa-mir-26b-5p, hsa-mir-182-5p, hsa-mir-34b-5p, hsa-mir-27a-3p, and hsa-mir-129-2-3p, which had more than three scores of degrees and top six betweenness. The TF regulator interaction with the hub genes is illustrated in **Figure 5F**. From **Figure 5F**, TFs including GATA2, PPARG, FOXC1, JUN, POU2F2, NFYA, STAT3, and SRF were found as significant hub TFs that had more than three scores of degrees and top eight betweenness as presented.

### 3.8 Correlation analysis between hub genes and infiltrating immune cells

**Figure 6:**
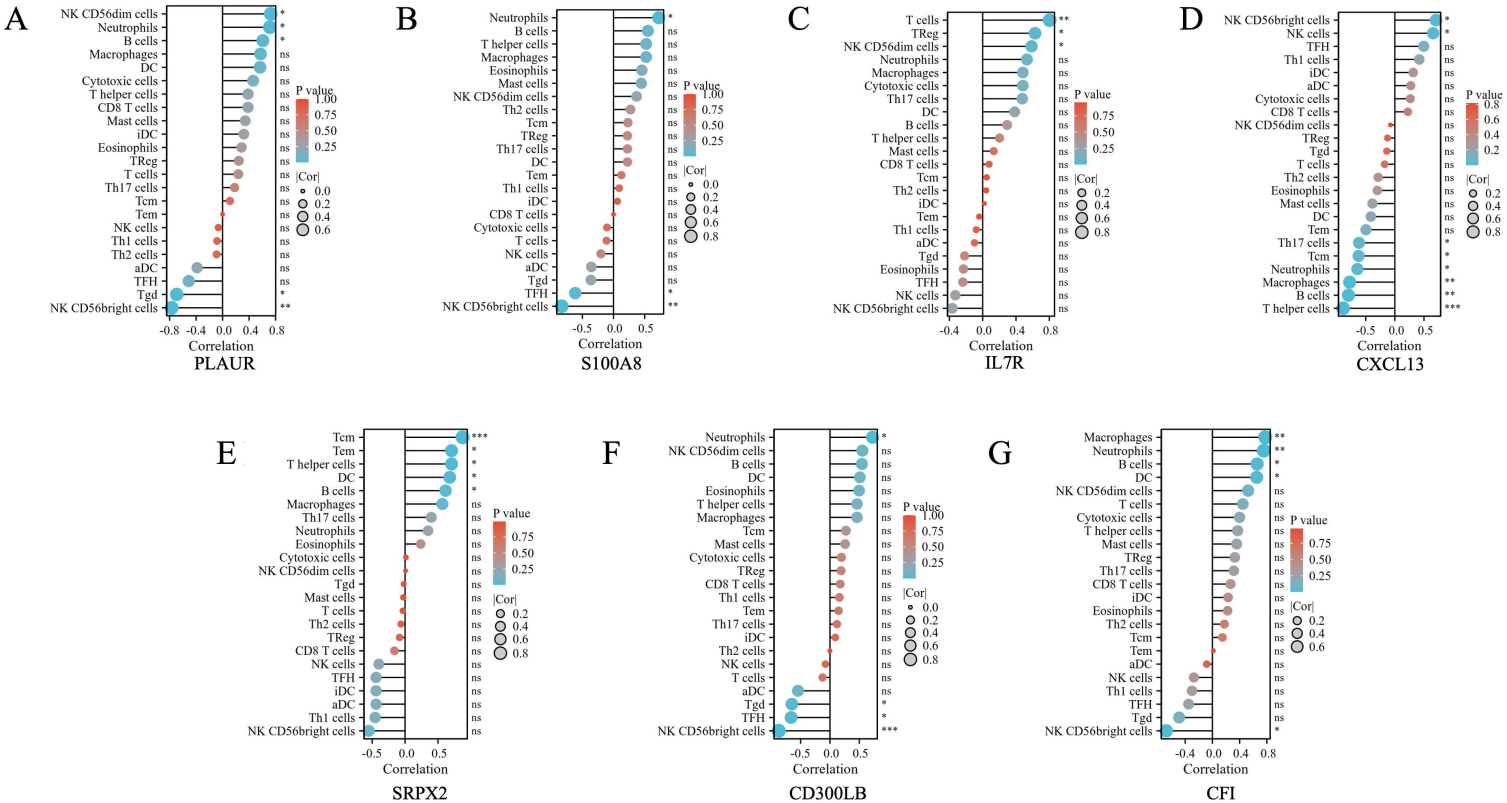
The correlation between differential immune infiltrating cells and the hub genes found in shared common DEGs of DPN mice and human samples. **(A)** The correlation between differential immune infiltrating cells and PLAUR. **(B)** The correlation between differential immune infiltrating cells and S100A8. **(C)** The correlation between differential immune infiltrating cells and IL7R. **(D)** The correlation between differential immune infiltrating cells and CXCL13. **(E)** The correlation between differential immune infiltrating cells and SRPX2. **(F)** The correlation between differential immune infiltrating cells and CD300LB. **(G)** The correlation between differential immune infiltrating cells and CFI. The color of the dots represents the *p*-value, and the size of the dots represents the strength of the correlation between genes and immune cells. *p < 0.05; **p < 0.01; *** *p* < 0.001. ns, not significant.

We subsequently performed a correlation analysis between hub genes and infiltrating immune cells to explore their potential relationships. As shown in **Figures 6A–G**, genes including PLAUR, S100A8, CD300LB, and CFI were positively correlated with neutrophils, while CXCL13 was negatively correlated with neutrophils (all *p* < 0.05). For NK CD56bright cells, PLAUR (*p* < 0.01), S100A8 (*p* < 0.01), CD300LB (*p* < 0.01), and CFI (*p* < 0.05) were highly negatively correlated with, while CXCL13 (*p* < 0.05) was highly positively correlated with.

### 3.9 Validation of DEGs by qRT-PCR

**Figure 7:**
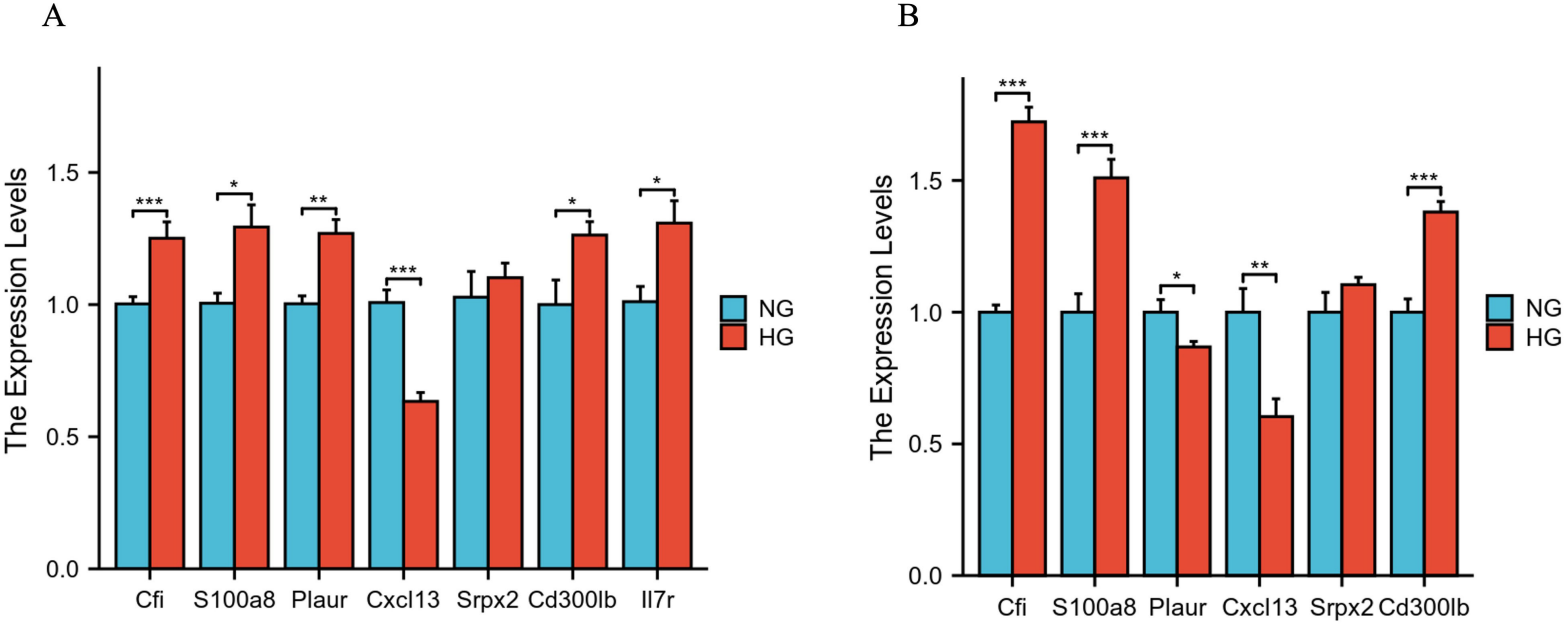
qRT-PCR validation of the hub genes in high- glucose-treated PC12 cells **(A)** and Schwann cells **(B)**. * *p* < 0.05; ** *p* < 0.01; *** *p* < 0.001.

The quantitative real-time PCR (qRT-PCR) was carried out to further validate hub genes obtained from microarray analysis using the high- glucose-treated PC12 cell model, which was a well-established peripheral nerve cell model previously used in the literature to mimic DPN nerve cells (26–28). As seen in **Figure 7A**, the expressions of Cfi (*p* < 0.001), S100a8 (*p* < 0.05), Plaur (*p* < 0.01), Cxcl13 (*p* < 0.001), Cd300lb (*p* < 0.001), and Il-7r (*p* < 0.05) were consistently changed in the HG group. Although the expressions of Srpx2 showed no statistically changes between the NG and HG group, there was an increasing trend in the HG group that was consistent with our previous findings.

Schwann cells were reported to have a critical role in the peripheral nervous system (29) and the high-glucose Schwann cell culture model was also a well-established cell model validated for DPN-like conditions in previous literature (30–33). Recent studies used the single-cell RNA-seq for peripheral nerve tissues isolated from DPN animals, and the results demonstrated that not only neurons but also Schwann cells played vital roles in the progression of DPN (34). We subsequently performed qRT-PCR on the high- glucose-treated Schwann cell model. As seen in **Figure 7B**, the expressions of Cfi (*p* < 0.001), S100a8 (*p* < 0.001), Cxcl13 (*p* < 0.01), and Cd300lb (*p* < 0.001) were significantly changed in the HG group and the changing trends were consistent with the previous bioinformatics findings. Similar to the results in the high- glucose-treated PC12 cell model, the expressions of Srpx2 showed no statistical changes between the NG and HG group in Schwann cells. Interestingly, the expressions of Plaur (*p* < 0.05) were significantly decreased in the HG group that was inconsistent with bioinformatics findings, which might be because nerve cells and glial cells behave differently. For the Il-7r gene, we found that expressions were very few in the Schwann cells, which consequently was not demonstrated.

## 4 Discussion

In light of advancements in gene sequencing technology, a substantial volume of gene chip data pertaining to DM and its associated complications has been deposited into online repositories (35), which has provided new insights for the diagnosis and treatment of DM and its complications, including DPN (9), diabetic kidney disease (DKD) (36), and diabetic cardiomyopathy (37). In this study, bioinformatics methods were employed to retrieve nerve datasets of DPN from the GEO database and dbGaP for a combined analysis. A total of 323 DEGs were identified in mouse DPN, which were further validated and intersected with the human DPN DEGs, revealing 13 shared DEGs. Subsequently, GO analysis, KEGG analysis, and PPI analysis were performed on these selected DEGs. Among them, seven genes, namely PLAUR, S100A8, IL7R, SRPX2, CD300LB, CFI, and CXCL13, were identified as core PPI sites that indicated the potential new targets in diagnosing and treating DPN.

The PLAUR gene, which was reported responsible for encoding the urokinase plasminogen activator receptor (uPAR) protein, exhibited primary expression in neutrophils, monocytes, and macrophages, playing a crucial role in various physiological pathways including the plasminogen activation pathway, inflammation, regulation of cell adhesion, migration, proliferation, and division (38). Previous research has indicated that uPAR, especially its soluble form, could serve as a diagnosis and prognosis biomarker in T1DM-related (39), T2DM-related (40), and DM-related complications such as diabetic cardiovascular diseases (41) and DKD (42). The SRPX2 gene, responsible for encoding a unique chondroitin sulfate proteoglycan, has been identified as playing a role in mediating seizure disorders, angiogenesis, and cellular adhesion (43). Furthermore, SRPX2 has been shown to act as a ligand for uPAR, contributing to the proteolysis of the extracellular matrix, angiogenesis, and endothelial cell remodeling (44). In our study, we found that both PLAUR and SRPX2 were upregulated, further suggesting the potential application of elevated uPAR levels as a future diagnosis and prognosis biomarker in DPN.

The S100A8 protein, a member of the S100 protein family, predominantly formed S100A8/A9 heterodimers in the body and functioned as an important pro-inflammatory factor. Upon binding to TLR4 or RAGE, the S100A8/A9 heterodimer acted as a critical cell activation factor, stimulating the secretion of pro-inflammatory cytokines by immune cells and facilitating the recruitment, aggregation, and adhesion of leukocytes. Recent studies have implicated S100A8/A9 in the pathogenesis of various diabetic complications, such as diabetic retinopathy (45), diabetic foot ulcers (46), and diabetic atherosclerosis (47). Lei et al. et al. observed a significant increase in the expression of S100A8 and S100A9 in DKD and suggested that targeting S100A8/A9 could be a promising therapeutic approach for DKD (48). In line with a previous report, we found that S100A8 gene was also upregulated, indicating the possible usage of inhibition of S100A8/A9 in the therapy of DPN in the future.

The gene IL-7R encoded a protein that was a member of the type I cytokine receptor family and found mainly expressed in lymphoid precursor cells (pre-L), B progenitor cells (pro-B), T cells, thymocytes, myeloid cells, and monocytes. This protein played a crucial role in the development and specialization of lymphocytes through its interaction with the ligand IL-7 (49). Research conducted by Paul et al. reported that patients with T1DM had lower monocyte IL-7R expression (50). Kevan et al. et al. utilized RN168, a monoclonal antibody targeting the IL7Rα, in patients of T1DM, and observed that RN168 selectively hindered the survival and function of memory T cells, while maintaining the populations of naive T cells and Tregs (51). In our study, we did not validate Il-7r expressions in high- glucose-treated Schwann cells because of the poor expression of Il-7r in Schwann cells. However, we observed a significant upregulation of IL-7R in the high- glucose-treated PC12 cells and the whole DPN nerve samples, and the expression was highly correlated with T cells, which indicated the possible usage of inhibition of IL-7R in T cells for treating DPN.

Complement Factor I, encoded by the CFI gene, served as an inhibitor of the complement system and played a role in the complement activation pathway. In the presence of cofactors, CFI was able to cleave C3b and C4b, thereby modulating immune responses and preventing excessive activation of the complement system. Previous reports found that CFI was increased in diabetic complications such as diabetic retinopathy (52) and diabetic cardiovascular diseases (53). Consistent with a previous report, we found that CFI gene was also upregulated in DPN and subsequently validated *in vitro*, which indicated that the immune response activation mediated by CFI was important in DPN pathogenesis.

CXC chemokines were a small class of peptide molecules that acted in conjunction with G protein-coupled receptors (GPCRs) to recruit immune cells involved in inflammatory responses. CXCL13, a CXC chemokine ligand, served as the primary regulator for directed chemotaxis of B cells, specifically binding and modulating the directed movement of B cells, participating in inflammatory responses. Sisi et al. et al. reported that CXCL13/CXCR5 signaling contributed to diabetes-induced tactile allodynia in the spinal cord of male mice (54). Hui et al. et al. found that overexpression of CXCL13 promoted the proliferation of bone marrow stromal cells’ *in vitro* high-glucose environment (55). In this study, a significant downregulation of CXCL13 was observed in the DPN group compared to the control group, suggesting a potential role for CXCL13 deficiency in the pathogenesis of DPN via inflammatory mechanisms. Conversely, upregulation of CXCL13 may hold therapeutic promise for the treatment of DPN.

Notably, in our study, both the DEGs in mice and humans were significantly enriched in immune response pathways using various enrichment analysis. Prior studies have suggested that heightened low-grade chronic inflammation was a significant factor in the pathogenesis of DPN. Elevated levels of inflammatory markers such as high-sensitivity C-reactive protein, IL-6, tumor necrosis factor-alpha (TNF-α), IL-1RA, and soluble intercellular adhesion molecule-1 showed strong correlations with the onset and progression of DPN (56). Thus, we conducted immune infiltration analysis to examine disparities in immune cell infiltration in human DPN. The findings suggested a significant higher proportion of neutrophils in DPN, as demonstrated in **Figure 3F**. Additionally, hub genes such as PLAUR, S100A8, CXCL13, CD300LB, and CFI were found to be significantly correlated with neutrophils, as shown in **Figure 6**. Neutrophils have been recognized as a key player in the innate immune system and acute inflammation, but emerging evidence suggested their active participation in chronic inflammatory processes and adaptive immune responses (57). Shahrabi et al. et al. conducted a meta-analysis investigating the association between neutrophil- to-lymphocyte ratio (NLR) and DPN, and the results showed that individuals with DPN exhibited elevated NLR levels compared to those without DPN (58). Li et al. et al. found that when NLR ≥2.66, the odds ratio was significantly higher for the risk of DPN, which indicated that NLR could be a predictive indicator in DPN (59).

As the third largest subset of lymphocytes, NK cells played a crucial role in the innate immune response and have been implicated in serving as a link between innate and adaptive immunity in the development of autoimmune diseases such as TIDM (60). Additionally, Seaward et al. observed that circulating NK cells in diabetic women exhibited altered tissue homing capabilities during and after pregnancy (61). NK cells could be divided into CD56 dim NK cells and CD56 bright NK cells based on the relative quantities of low-affinity FcγR CD16 and adhesion molecule CD56. The CD56 bright NK cells were known to predominantly produce various cytokines, including IL-10, interferon-γ, tumor necrosis factor-α, and granulocyte-macrophage colony-stimulating factor, which played a regulatory role in the function of dendritic cells, monocytes, and T cells (62). In our study, we found that the proportion of CD56 bright NK cells in human DPN samples was significantly higher than that in the control group. In addition, the hub genes including PLAUR, S100A8, CXCL13, CD300LB, and CFI were significantly correlated with CD56 bright NK cells, which suggested that modifying expression of those above hub genes might influence cell subtypes of NK and facilitate the production of specific chemokines to modulate the immune function status of the body, thus subsequently influencing the pathogenesis of DPN.

There were several limitations in this study. Firstly, the validation of the hub genes and the exploration about the TFs and microRNAs related to hub genes were inadequate, particularly due to the absence of *in vivo* experiments. Therefore, future research should focus on conducting functional validation and investigating the molecular mechanisms to confirm the biological significance of key genes and the upstream regulatory mechanisms of key genes and downstream targets associated with DPN. Secondly, the diversity and complexity of biological systems posed challenges to bioinformatics methods as different species, genomes, and biological processes might have unique characteristics and patterns. Due to data noise, incomplete data preprocessing, and algorithm limitations, it might lead to erroneous discoveries or omissions of real biological patterns to reveal the real relationships between immune regulation and hub genes. More specific analysis methods and tools need to be developed and performed in the future. Ultimately, the retrospective data mining restricted the adequate acquisition of clinical information pertaining to human nerve tissue samples. Additional studies involving more clinical details were required for further validation.

## 5 Conclusions

In this study, we utilized bioinformatics analysis to identify DEGs in multiple DPN datasets from mice and humans, revealing common shared DEGs between the two species. Subsequently, seven hub genes were identified through PPI analysis and mostly confirmed through *in vitro* experiments. Additionally, key TFs and microRNAs interacting with the hub genes were identified. Furthermore, through functional enrichment and pathway analysis of DEGs, the importance of dysregulated immune response in the pathogenesis of DPN was revealed. Thus, we examined immune cell infiltration in human DPN samples and explored the relationship between hub genes and immune cells. Ultimately, these findings hopefully provided new research targets and insights for the diagnosis and treatment of DPN.

## Data Availability

The original contributions presented in the study are included in the article/**Supplementary Material**. Further inquiries can be directed to the corresponding authors.
