# Supplementary material for: Identification of key genes and immune infiltration of diabetic peripheral neuropathy in mice and humans based on bioinformatics analysis
Source: Front Endocrinol (Lausanne). 2024 Nov 18;15:1437979. doi: 10.3389/fendo.2024.1437979 (PMC11608978; doi:10.3389/fendo.2024.1437979)
Supplement: Supplementary file 1 [file Table1.docx]

**Table S1** The primer sequences used for PCR

| Primer name | Species | Primer sequence |
| --- | --- | --- |
| β-actin-F | Rattus norvegicus | CTGTGTGGATTGGTGGCTCT |
| β-actin-R | Rattus norvegicus | AGCTCAGTAACAGTCCGCCT |
| Plaur-F | Rattus norvegicus | GCACAGCAGGTTTCCATAGC |
| Plaur-R | Rattus norvegicus | ATTGGAAGCCATTCGGTGGT |
| S100a8-F | Rattus norvegicus | AGGCCTTGAGCAACGTCATT |
| S100a8-R | Rattus norvegicus | GGGCACTCAGTAGTGACCAT |
| Cxcl13-F | Rattus norvegicus | CCTCCAGGCCACGGTATTCT |
| Cxcl13-R | Rattus norvegicus | AGCTTTCTTGGCCTTGGTCC |
| Srpx2-F | Rattus norvegicus | GGTAACACTTCGGGGTCCAG |
| Srpx2-R | Rattus norvegicus | GGACAGCGTCTCACTTGTACT |
| Cd300lb-F | Rattus norvegicus | TCTCAGTTTCCCAGGGTGCC |
| Cd300lb-R | Rattus norvegicus | ACATTGCACAGTCACCGACC |
| Cfi-F | Rattus norvegicus | GTTGGCAGTTCAGGGAGTGA |
| Cfi-R | Rattus norvegicus | TCTTTCTTGCCCGGGTGTTT |

**Table S2** Ontological analysis of common DEGs in DPN mice

| Category | GO ID | Description | P Value | Genes |
| --- | --- | --- | --- | --- |
| Biological  Process | GO:0002683 | negative regulation of immune system process | 1.6275E-15 | Thy1, A2m, Pla2g2d, Ctsg, Arg1, Il7r, Gpnmb, Lgals3, Dusp10, Trem2, Thbs1, Pdcd1lg2, Zc3h12d Cd22, Cd84, Ccl2, Lpxn, Otop1, Havcr2, Cd200r1, Mmp12, Cd300a, Serpinb9b, Cd68, Cnr2, Vsig4, Tmem178, Ptpn22, Stap1, Npy |
|  | GO:0022407 | regulation of cell-cell adhesion | 1.5323E-12 | Thy1, Rap1gap, Pcdh8, Pla2g2d, Cxcl13, Ctsg, Il1rn, Cd1d1, Arg1, Il7r, Dock8, Gpnmb, Lgals3, Adam8, Lep, Dusp10, Pdcd1lg2, Zc3h12d, Plaur, Ccl2, Havcr2, Cd300a, Ptger3, Itgb2, Vsig4, Ptpn22, Card11 |
|  | GO:0050900 | leukocyte migration | 4.2064E-12 | Thy1, Mmp9, Cxcl13, Ctsg, F7, Dock8, Serpine1, Lgals3, Adam8, Lep, S100a8, Trem2, Thbs1, Ccl2, Cd200r1, C3ar1, Cd300a, Ptger3, Itgb2, Ccl22, Ccl3, Cnr2, Ptpn22, Stap1 |
|  | GO:0007159 | leukocyte cell-cell adhesion | 4.9658E-12 | Thy1, Pla2g2d, Ctsg, Olr1, Cd1d1, Arg1, Il7r, Dock8, Gpnmb, Lgals3, Adam8, Lep, Dusp10, S100a8, Thbs1, Pdcd1lg2, Zc3h12d, Ccl2, Havcr2, Cd300a, Itgb2, Vsig4, Ptpn22, Card11 |
|  | GO:0044772 | mitotic cell cycle phase transition | 5.4042E-12 | Ubd, Hmga2, Gpnmb, Cdk1, Cdk18, Brca1, Zc3h12d, Dtl, Cdca5, Ccl2, Ccnb2, Spdl1, Birc5, Ccnb1, Ccne1, Cdc25c, Bub1, Plk1, Aurkb, Rrm2, Stil, Ndc80, Ccna2, Clspn, Ttk |
|  | GO:1901990 | regulation of mitotic cell cycle phase transition | 2.7655E-11 | Ubd, Hmga2, Gpnmb, Cdk1, Brca1, Zc3h12d, Dtl, Cdca5, Ccl2, Spdl1, Birc5, Ccnb1, Cdc25c, Bub1, Plk1, Aurkb, Rrm2, Stil, Ndc80, Clspn, Ttk |
|  | GO:1901991 | negative regulation of mitotic cell cycle phase transition | 5.5003E-11 | Hmga2, Gpnmb, Cdk1, Brca1, Zc3h12d, Dtl, Ccl2, Spdl1, Birc5, Ccnb1, Bub1, Plk1, Aurkb, Ndc80, Clspn, Ttk |
|  | GO:0002274 | myeloid leukocyte activation | 1.0847E-10 | Ctsg, Ubd, Cd1d1, Dcstamp, Trem2, Thbs1, Cd84, Lat2, Havcr2, Cd300a, Itgb2, Clec4d, Sphk1, Cnr2, Myo1f, Mmp8, Stap1, Npy, Cd300lb |
|  | GO:1903037 | regulation of leukocyte cell-cell adhesion | 1.6993E-10 | Thy1, Pla2g2d, Ctsg, Cd1d1, Arg1, Il7r, Dock8, Gpnmb, Lgals3, Adam8, Lep, Dusp10, Pdcd1lg2, Zc3h12d, Ccl2, Havcr2, Cd300a, Itgb2, Vsig4, Ptpn22, Card11 |
|  | GO:1901988 | negative regulation of cell cycle phase transition | 2.1684E-10 | Susd2, Hmga2, Gpnmb, Cdk1, Brca1, Zc3h12d, Dtl, Ccl2, Spdl1, Birc5, Ccnb1, Bub1, Plk1, Aurkb, Stil, Ndc80, Clspn, Ttk |
| Cellular  Component | GO:0000775 | chromosome, centromeric region | 3.5858E-08 | Nek2, Spc25, Spdl1, Birc5, Ccnb1, Spc24, Bub1, Plk1, Aurkb, Esco2, Ndc80, Top2a, Mis18bp1, Cenph, Ttk |
|  | GO:0000776 | kinetochore | 8.1592E-08 | Nek2, Spc25, Spdl1, Birc5, Ccnb1, Spc24, Bub1, Plk1, Aurkb, Ndc80, Cenph, Ttk |
|  | GO:0000779 | condensed chromosome, centromeric region | 1.6815E-07 | Nek2, Spc25, Spdl1, Birc5, Ccnb1, Spc24, Bub1, Plk1, Aurkb, Ndc80, Cenph, Ttk |
|  | GO:0000793 | condensed chromosome | 2.1502E-06 | Nek2, Spc25, Brca1, Spdl1, Birc5, Ccnb1, Spc24, Ncaph, Bub1, Plk1, Aurkb, Ndc80, Top2a, Cenph, Ttk |
|  | GO:0098687 | chromosomal region | 4.5621E-06 | Nek2, Spc25, Spdl1, Birc5, Ccnb1, Spc24, Bub1, Plk1, Aurkb, Esco2, Ndc80, Top2a, Mis18bp1, Cenph, Ttk |
|  | GO:0098793 | presynapse | 9.2103E-06 | Slc6a2, Cdh9, Slc4a10, Adcy8, Syt9, Syt4, Sh3gl2, Snap91, Cntnap2, Slc6a17, Syngr3, Slc5a7, Ccl2, Fosl1, Sphk1, Igsf21, Slc18a1, Npy |
|  | GO:0000940 | outer kinetochore | 1.2676E-05 | Spdl1, Ccnb1, Plk1, Ndc80 |
|  | GO:0016324 | apical plasma membrane | 1.4923E-05 | Thy1, Slc4a10, Adcy8, Rab27b, Kcnj10, Slc5a7, Oxtr, Abcb4, S100g, Atp6v0d2, Gpihbp1, Cd300lg, Birc5, Hvcn1, Slc22a12 |
|  | GO:0062023 | collagen-containing extracellular matrix | 3.3774E-05 | Col2a1, Mmp9, Pcolce2, Angpt4, Cela1, Angptl3, Ncan, Mfge8, Gsto1, Ltbp2, Serpine1, Lgals3, Thbs1, Angptl4, Srpx2 |
|  | GO:1905286 | serine-type peptidase complex | 4.7017E-05 | F7, Cfi, F10, Plaur |
| Molecular  Function | GO:0016411 | acylglycerol O-acyltransferase activity | 1.4057E-06 | Mogat1, Mogat2, Lpgat1, Dgat2, Pnpla3 |
|  | GO:0005539 | glycosaminoglycan binding | 1.5814E-06 | Pcolce2, Pla2g2d, Cxcl13, Ctsg, Angptl3, Ncan, Ltbp2, Gpnmb, Trem2, Thbs1, Ccl2, Igfals, Stab2 |
|  | GO:0019838 | growth factor binding | 6.4088E-06 | Col2a1, A2m, Cxcl13, Il1rn, Igfbp3, Ltbp2, Thbs1, Klb, Igfals, Srpx2 |
|  | GO:0001786 | phosphatidylserine binding | 8.3917E-06 | Syt9, Syt4, Mfge8, Trem2, Thbs1, Cd300a, Gsdma |
|  | GO:0008201 | heparin binding | 1.6156E-05 | Pcolce2, Pla2g2d, Cxcl13, Ctsg, Angptl3, Ltbp2, Gpnmb, Thbs1, Ccl2, Igfals |
|  | GO:0071813 | lipoprotein particle binding | 1.8485E-05 | Pon1, Gpihbp1, Trem2, Thbs1, Stab2 |
|  | GO:0071814 | protein-lipid complex binding | 1.8485E-05 | Pon1, Gpihbp1, Trem2, Thbs1, Stab2 |
|  | GO:0072341 | modified amino acid binding | 2.3607E-05 | Syt9, Syt4, Mfge8, Trem2, Thbs1, Cd300a, Ltc4s, Gsdma |
|  | GO:0050839 | cell adhesion molecule binding | 3.0672E-05 | Thy1, Cdh9, Cdh10, Cd1d1, Tenm4, Angptl3, Mfge8, Itgax, Gpnmb, Adam8, Thbs1, Cd200r1, Itgb2 |
|  | GO:0008237 | metallopeptidase activity | 3.3431E-05 | Ece2, Adamts16, Cpb1, Mmp9, Cpa3, Cpa2, Adam8, Trabd2b, Mmp12, Mmp8 |
| KEGG_PATHWAY | mmu04610 | Complement and coagulation cascades | 2.9358E-07 | A2m, F5, F7, Itgax, Cfi, Serpine1, F10, Plaur, C3ar1, Itgb2, Vsig4 |
|  | mmu04115 | p53 signaling pathway | 2.0716E-05 | Igfbp3, Serpine1, Cdk1, Thbs1, Ccnb2, Ccnb1, Ccne1, Rrm2 |
|  | mmu04110 | Cell cycle | 3.5497E-05 | Cdk1, E2f2, Ccnb2, Ccnb1, Ccne1, Cdc25c, Bub1, Plk1, Ccna2, Ttk |
|  | mmu04914 | Progesterone-mediated oocyte maturation | 0.00012239 | Adcy8, Cdk1, Ccnb2, Ccnb1, Cdc25c, Bub1, Plk1, Ccna2 |
|  | mmu04114 | Oocyte meiosis | 0.00079516 | Adcy8, Cdk1, Ccnb2, Ccnb1, Ccne1, Cdc25c, Bub1, Plk1 |
|  | mmu04080 | Neuroactive ligand-receptor interaction | 0.00156188 | Adrb3, Agtr1b, Lepr, Ctsg, Oxtr, Gabrr2, Chrna2, Tshr, Mc2r, Lep, Agtr2, C3ar1, Ptger3, Cnr2, Npy |
|  | mmu04371 | Apelin signaling pathway | 0.00177517 | Gng4, Agtr1b, Adcy8, Ucp1, Myl4, Serpine1, Pik3r5, Sphk1 |
|  | mmu04923 | Regulation of lipolysis in adipocytes | 0.00229724 | Adrb3, Adcy8, Tshr, Ptger3, Npy |
|  | mmu04972 | Pancreatic secretion | 0.00257896 | Cpb1, Adcy8, Rab27b, Pla2g2d, Cpa3, Cpa2, Tpcn2 |
|  | mmu04614 | Renin-angiotensin system | 0.00296827 | Agtr1b, Cpa3, Ctsg, Agtr2 |

Top 10 terms of each category are listed.

**Table S3** GSEA in human DPN samples.

| ID | setSize | enrichmentScore | NES | pvalue | p.adjust | qvalue |
| --- | --- | --- | --- | --- | --- | --- |
| REACTOME_FCGR3A_MEDIATED_IL10_SYNTHESIS | 95 | 0.8843550 | 3.134374 | 1e-10 | 9.96e-09 | 9.56e-09 |
| REACTOME_SIGNALING_BY_THE_B_CELL_RECEPTOR_BCR | 166 | 0.8202495 | 3.139684 | 1e-10 | 9.96e-09 | 9.56e-09 |
| REACTOME_FCGAMMA_RECEPTOR_FCGR_DEPENDENT_PHAGOCYTOSIS | 143 | 0.8386550 | 3.166825 | 1e-10 | 9.96e-09 | 9.56e-09 |
| REACTOME_FCERI_MEDIATED_NF_KB_ACTIVATION | 136 | 0.8541427 | 3.180674 | 1e-10 | 9.96e-09 | 9.56e-09 |
| REACTOME_PARASITE_INFECTION | 116 | 0.8723060 | 3.189050 | 1e-10 | 9.96e-09 | 9.56e-09 |

**Table S4** Ontological analysis of DEGs shared in mice and human

| Ontology | ID | Description | GeneRatio | BgRatio | pvalue | p.adjust | zscore |
| --- | --- | --- | --- | --- | --- | --- | --- |
| BP | GO:0002544 | chronic inflammatory response | 2/13 | 17/18800 | 5.97e-05 | 0.0194 | 0.0000000 |
| BP | GO:0051965 | positive regulation of synapse assembly | 2/13 | 63/18800 | 0.0008 | 0.0826 | 0.0000000 |
| BP | GO:0022409 | positive regulation of cell-cell adhesion | 3/13 | 291/18800 | 0.0009 | 0.0826 | 0.5773503 |
| BP | GO:0042742 | defense response to bacterium | 3/13 | 364/18800 | 0.0018 | 0.0826 | 0.5773503 |
| MF | GO:0019838 | growth factor binding | 2/13 | 139/18410 | 0.0042 | 0.0921 | 0.0000000 |
| MF | GO:0050786 | RAGE receptor binding | 1/13 | 10/18410 | 0.0070 | 0.0921 | 1.0000000 |
| MF | GO:0035325 | Toll-like receptor binding | 1/13 | 12/18410 | 0.0084 | 0.0921 | 1.0000000 |
| MF | GO:0036041 | long-chain fatty acid binding | 1/13 | 15/18410 | 0.0105 | 0.0921 | 1.0000000 |
| KEGG | hsa04610 | Complement and coagulation cascades | 2/6 | 85/8164 | 0.0016 | 0.0235 | 1.4142136 |
