# Supplementary material for: Identification of key genes and immune infiltration of diabetic peripheral neuropathy in mice and humans based on bioinformatics analysis
Source: Front Endocrinol (Lausanne). 2024 Nov 18;15:1437979. doi: 10.3389/fendo.2024.1437979 (PMC11608978; doi:10.3389/fendo.2024.1437979)
Supplement: Supplementary file 2 [file DataSheet1.docx]

**
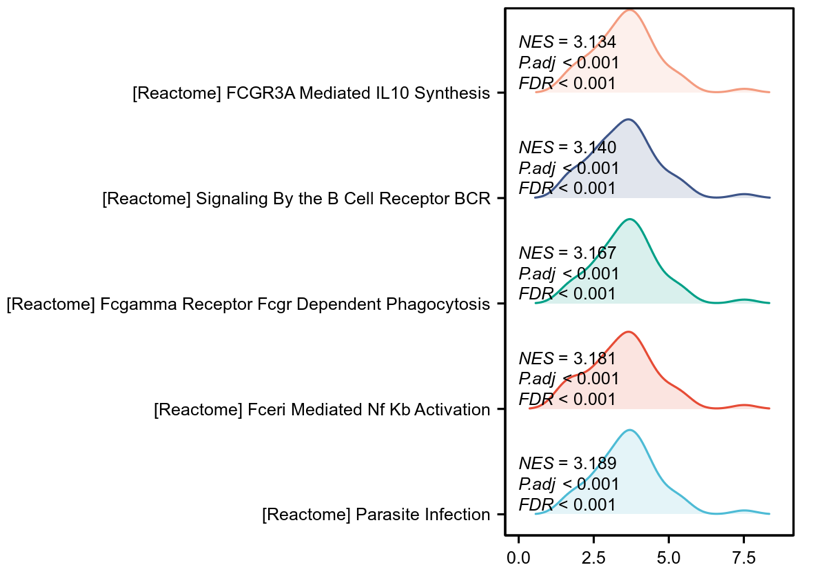
**

**Figure S1** GSEA in human DPN samples

**
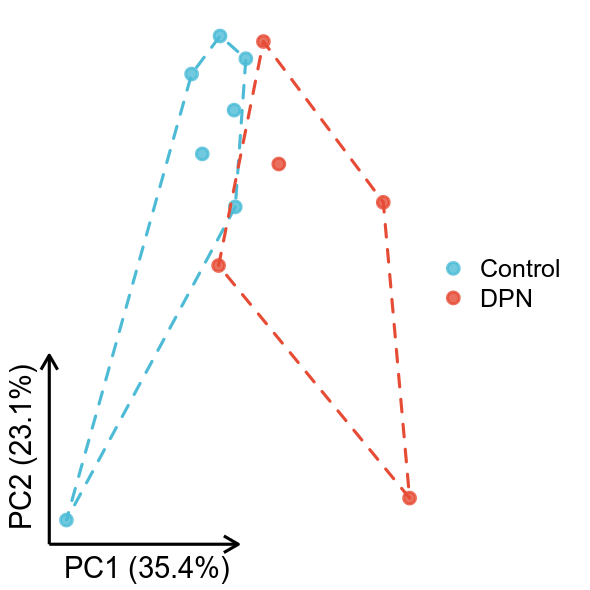
**

**Figure S2** PCA cluster plot of immune cell infiltration between control and DPN samples.


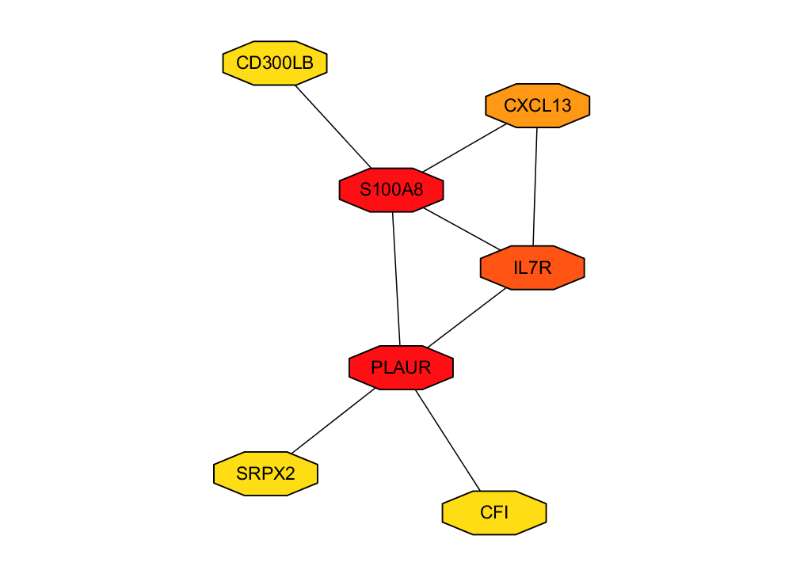


**Figure S3** PPI network of the shared DEGs between human and mice DPN generated by Cytoscape.


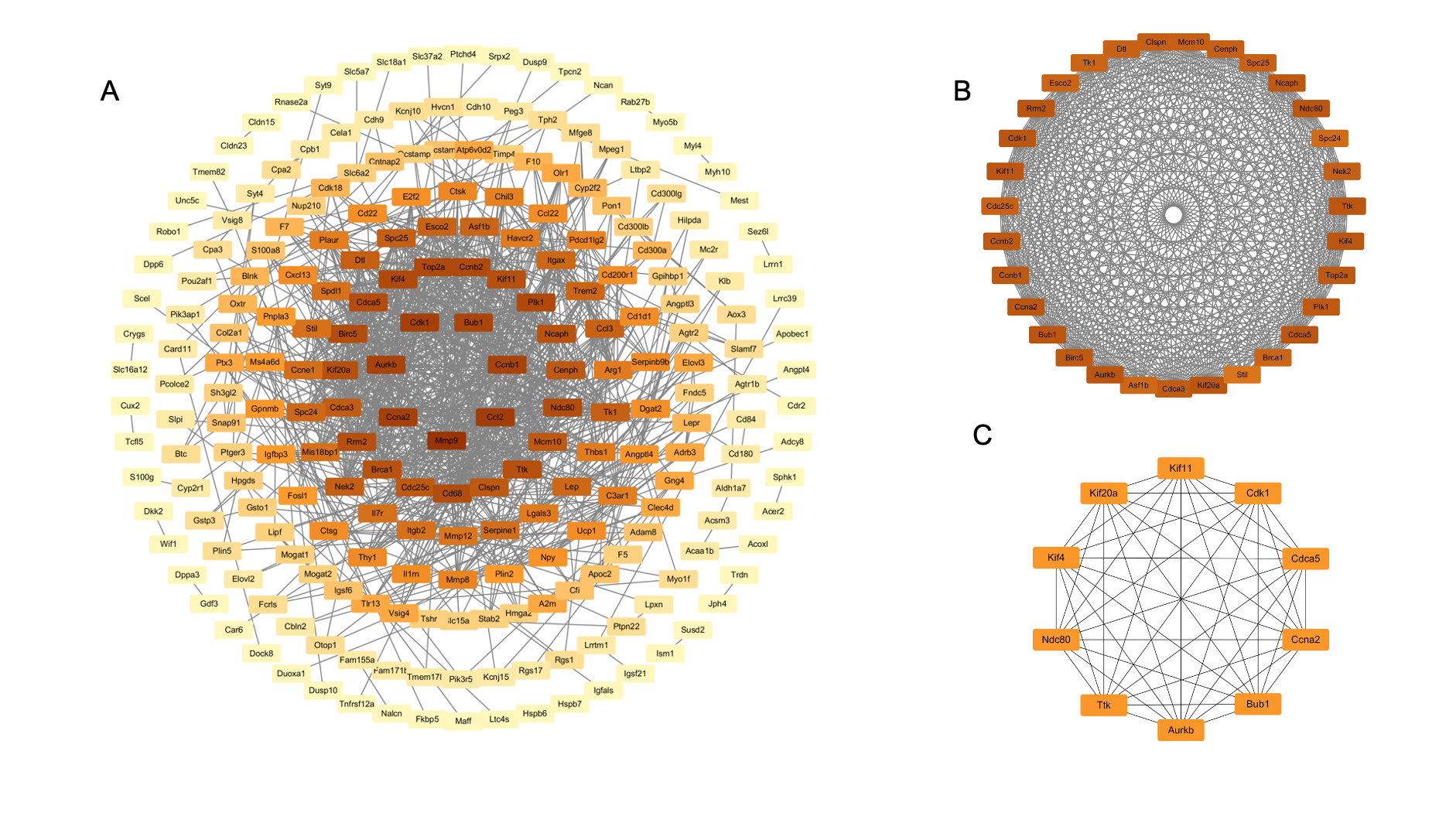


**Figure S4** Explorations of hub genes in mice DPN. (A) PPI network of mice common DEGs generated by Cytoscape. (B) The core subnetwork generated by MCODE. (C) Top 10 genes in the network selected by MCC.
